# Evaluation of Naturalistic Driving Behavior Using In-Vehicle Monitoring Technology in Preclinical and Early Alzheimer’s Disease

**DOI:** 10.3389/fpsyg.2020.596257

**Published:** 2020-10-27

**Authors:** Jennifer D. Davis, Ganesh M. Babulal, George D. Papandonatos, Erin M. Burke, Christopher B. Rosnick, Brian R. Ott, Catherine M. Roe

**Affiliations:** ^1^Department of Psychiatry and Human Behavior, Warren Alpert Medical School of Brown University, Providence, RI, United States; ^2^Neuropsychology Program, Department of Psychiatry, Rhode Island Hospital, Providence, RI, United States; ^3^Department of Neurology, Charles F. and Joanne Knight Alzheimer’s Disease Research Center, Washington University School of Medicine, St. Louis, MO, United States; ^4^Department of Biostatistics, Brown University, Providence, RI, United States; ^5^Department of Neurology, Rhode Island Hospital, Warren Alpert Medical School of Brown University, Providence, RI, United States

**Keywords:** driving, Alzheimer’s disease, naturalistic, technology, preclinical Alzheimer’s disease, driving mobility

## Abstract

Cognitive impairment is a significant risk factor for hazardous driving among older drivers with Alzheimer’s dementia, but little is known about how the driving behavior of mildly symptomatic compares with those in the preclinical, asymptomatic phase of Alzheimer’s disease (AD). This study utilized two in-car technologies to characterize driving behavior in symptomatic and preclinical AD. The goals of this pilot study were to (1) describe unsafe driving behaviors in individuals with symptomatic early AD using G-force triggered video capture and (2) compare the driving habits of these symptomatic AD drivers to two groups of cognitively normal drivers, those with and those without evidence of cerebral amyloidosis (CN/A+ and CN/A−) using a global positioning system (GPS) datalogger. Thirty-three drivers (aged 60+ years) were studied over 3 months. G-force triggered video events captured instances of near-misses/collisions, traffic violations, risky driver conduct, and driving fundamentals. GPS data were sampled every 30 s and all instances of speeding, hard braking, and sudden acceleration were recorded. For the early AD participants, video capture identified driving unbelted, late response, driving too fast for conditions, traffic violations, poor judgment, and not scanning intersections as the most frequently occurring safety errors. When evaluating driving using the GPS datalogger, hard breaking events occurred most frequently on a per trip basis across all three groups. The CN/A+ group had the lowest event rate across all three event types with lower instances of speeding. Slower psychomotor speed (Trail Making Part A) was associated with fewer speeding events, more hard acceleration events, and more overall events. GPS tracked instances of speeding were correlated with total number of video-captured near-collisions/collisions and driving fundamentals. Results demonstrate the utility of electronic monitoring to identify potentially unsafe driving events in symptomatic and preclinical AD. Results suggest that drivers with preclinical AD may compensate for early, subtle cognitive changes by driving more slowly and cautiously than healthy older drivers or those with cognitive impairment. Self-regulatory changes in driving behavior appear to occur in the preclinical phase of AD, but safety concerns may not arise until symptoms of cognitive impairment emerge and the ability to self-monitor declines.

## Introduction

Older drivers with cognitive impairment are at high risk for unsafe driving, carrying a relative crash risk of 2–5 times higher compared to matched controls (Marshall, 2008). Alzheimer’s disease (AD), one of the most prevalent diseases affecting cognition in older adults (Alzheimer’s Association, 2019), adversely impacts driving. Individuals with AD are at increased risk for failing a road test with disease progression (Duchek et al., 2003; Ott et al., 2008), make more safety errors when driving in their own environment compared to cognitively normal older adults (Davis et al., 2012), and are at greater risk for crashes (Drachman and Swearer, 1993). Despite their increased risk, drivers with AD continue to drive during their disease course but may modify their behavior by reducing driving in complex situations (Festa et al., 2013; Molnar et al., 2013). This includes driving without passengers, during daytime hours, good weather, light traffic, and residential rather than commercial environments. Festa et al. (2013) Despite behavioral modification, however, most drivers with AD eventually need to cease driving due to progressive cognitive and functional decline (Connors et al., 2018).

Little is known about how driving is affected early in the disease process or the pathological process underlying that decline. AD begins decades (∼2–3) before overt expression of cognitive symptoms as beta amyloid, the pathological marker of AD, begins to accumulate in the brain. The presence of cerebral amyloid has been directly associated with driving. Specifically, several postmortem studies of the brains of older drivers who were killed in motor vehicle crash (MVCs) have found that many had the neuropathologic changes of AD but had never been diagnosed (Johansson et al., 1997; Viitanen et al., 1998; Kibayashi and Shojo, 2002; Gorrie et al., 2007).

The development of biomarkers for AD has allowed for *in vivo* studies of amyloid deposition and driving behavior. Traffic violations and accidents over the 3 years prior to brain imaging was strongly related to accumulating amyloid on positron emission tomography [PET] scans, even in individuals not yet displaying measurable cognitive impairments resulting from the disease (i.e., preclinical AD) (Ott et al., 2017b). More abnormal levels of cerebral amyloid detected using Pittsburgh compound B radiotracer via PET predicted poorer performance on a standardized road test among cognitively normal older adults (Roe et al., 2017). Using naturalistic methodology, real world driving behavior appears to change even in the preclinical phase of the disease with amyloid positive older adults driving to fewer places/unique destinations, traveling fewer days, and taking fewer trips compared to amyloid negative same-aged peers. Furthermore, those with preclinical AD had fewer trips with any aggressive behaviors and showed a greater decline across a 2.5-year follow-up period in the number of days driving per month and number of trips taken (Roe et al., 2019).

## Current Study

To date, there is little data examining the spectrum of age-related driving behavior ranging from normal cognition to preclinical AD to symptomatic AD. Using a convenience sample of older drivers, the goal of this pilot study was to describe naturalistic driving behavior among these three groups using in-vehicle video and global positioning system (GPS) technologies. The first aim was to describe the types of hazardous driving errors captured by video technology in a subset of the sample of older adult drivers with early AD. The second aim was to compare the driving behaviors of drivers with early AD to two groups of cognitively normal (CN) drivers, those with evidence of brain amyloid (preclinical AD; CN/A+) to healthy adults without evidence of brain (CN/A−) over 3 months of naturalistic driving.

## Materials and Methods

### Participants

Symptomatic AD drivers (*n* = 11) were recruited from a multidisciplinary outpatient memory clinic in Rhode Island. All participants underwent a diagnostic evaluation by a neurologist at the Center. Neurological examination results were judged to be normal for age or consistent with AD. For inclusion, Mini-Mental State Examination (MMSE) (Folstein et al., 1975) scores were <28 and Clinical Dementia Rating (Morris, 1993) (CDR) scores were categorized as CDR = 0.5 or 1, indicating questionable or mild dementia. It is well established that CDR 0.5 is equivalent to mild cognitive impairment (Morris et al., 2001). Participants met diagnostic criteria for possible or probable AD based on NINCDS-ADRDA criteria (McKhann et al., 1984). Patients were on a stable dose of a cholinesterase inhibitor for 6 weeks, if prescribed. The amyloid status was known only for a subset of the cognitively impaired participants, as amyloid imaging was not a standard part of the original study protocol. Amyloid imaging was obtained within 18 months of study entry (*M* = 333 days; range = 56–511 days). Amyloid imaging results are presented in Table 1 to add to the clinical characterization of the early AD group. This subset of participants underwent amyloid PET imaging using the radiotracer ^18^F-Florbetapir (Clark et al., 2011) as part of their participation in other clinical research studies. An established standardized uptake value ratio (SUVR) threshold of >1.19 was used to indicate amyloid PET positivity (Clark et al., 2012; Johnson et al., 2013). All scans were read by two clinical neuroradiologists who gave also gave a clinical read of the scan. One participant had a SUVR threshold of 1.16 but had a positive clinical read. That participant was considered to be amyloid positive in this study. The sample was further characterized by apolipoprotein (ApoE) genotype, a known risk factor for AD if the ε4 allele is present. Of the eight participants with ApoE genotyping completed, 50% possessed the ε4 allele.

**TABLE 1:** Demographic characteristics of participants.

	**CDR 0 (CN/A−) (*n* = 11) (%) or M (SD)**	**CDR 0 (CN/A+) (*n* = 11) N (%) or M (SD)**	**CDR.5/1 (AD) (*n* = 11) N (%) or M (SD)**	**Statistic**	***p***
Women, *N*	5 (45%)	5 (45%)	5 (45%)		
White, *N*	10 (91%)	11 (100%)	11 (100%)		
MCI/CDR.5, N	0 (0%)	0 (0%)	9 (82%)		
MMSE (total)	29.18 (1.67)	29.09 (1.30)	25.18 (3.84)	*F* = 9.66	0.001*
Age, years	73.33 (5.21)	73.71 (5.12)	72.88 (6.84)	*F* = 0.56	0.95
Education (years)	17.45 (1.97)	16.55 (1.86)	15.45 (3.36)	*F* = 1.78	0.19

*Implies difference between CDR 0 vs. CDR.5/1.CN/A−, cognitively normal/amyloid negative; CN/A+, cognitively normal/amyloid positive; AD, Alzheimer’s disease; CDR, Clinical Dementia Rating Scale; MCI, mild cognitive impairment.

All participants were >60 years of age, English speaking with a valid driver’s license. Exclusion criteria included ophthalmologic, physical, or neurologic disorders other than dementia that impair their driving abilities, visual acuity worse than 20/40 in best eye using distance vision measured by wall chart, homonymous hemianopia or bitemporal hemianopia, musculoskeletal disorders causing major physical handicaps, history of alcohol or substance abuse by DSM V criteria within the past year, had used sedating medications that impair level of consciousness or attention, had a language impairment that would interfere with the ability to participate in the study, or had a previous road test evaluation or opinion of caregiver or health professional that they were unsafe to drive. Study protocols were approved by the Rhode Island Hospital Institutional Review Board, and all participants provided written informed consent that was also signed by a study partner.

Since beta-amyloid is the primary driver and earliest marker of AD pathogenesis and cascade, it was selected as the main biomarker (Jack et al., 2018). A group of cognitively normal drivers were selected from participants enrolled in a longitudinal study assessing preclinical AD and driving performance (R01 AG043434) at Washington University School of Medicine in St. Louis and matched for age and gender to the early AD group. All participants were cognitively normal (CDR = 0), ≥65 years old, had a valid driver’s license, drove at least once per week, and had *in vivo* imaging of amyloid using PET with either Pittsburgh compound B (PIB) or florbetapir AV45 radiotracer to confirm group membership. PET imaging was selected if it occurred 2 years before or 6 months after the installation date of datalogger in the participant’s vehicle. Eleven participants were selected with amyloid negative scans and 11 with evidence of amyloid based on centiloid values (Su et al., 2015). Accepted cut-offs of centiloids were based on the mean cortical SUVR with partial volume correction via regional spread function (RSF) [PIB MCSUVR RSF ≥ 16.4 and AV45 MCSUVR RSF ≥ 20.6] (Su et al., 2015, 2018, 2019). The amyloid negative group had 27.3% the ApoE ε4 allele carriers, and 63.6% were ApoE ε4 allele carriers in the amyloid positive group. The participant’s vehicle had to be manufactured in the year 1996 or newer in order to have access to the onboard diagnostic port (OBDII). Study protocols were approved by the Washington University Human Research Protection Office, and written informed consent was obtained from all participants.

### Study Procedures

#### Cognition

At study enrollment, all participants completed cognitive measures, including a global measure of cognition (MMSE) and a task of psychomotor speed and set shifting (Trail Making Parts A and B, respectively) (Reitan, 1956). Trail making was selected because it has been shown to be related to naturalistic driving errors (Papandonatos et al., 2015). Time in seconds to complete the tasks were used in data analyses. Higher scores on Trails A and B reflect worse performance (i.e., slower time).

#### Technology

Vehicles were equipped with two forms of technology, an event-based video recording system (Drivecam)^®^ was equipped for CDR > 0 drivers and a GPS datalogger was equipped for all drivers. Because these results reflect the combination of drivers from two different parent studies, only the vehicles of the mild AD group were equipped with the camera system. All three groups had the GPS datalogger installed to capture naturalistic driving behavior.

The *DriveCam* video camera is a palm-sized, exception-based video event recorder that was mounted in a bracket secured to the windshield behind the rearview mirror with an adhesive similar to what holds the rearview mirror in place. This system is a validated method for detecting and evaluating driving safety errors in AD (Ott et al., 2017a). Camera views were the forward roadway and the driver in the vehicle interior. Once installed, the camera continuously captured video and temporarily saved the previous several seconds in a video buffer. If the device was not triggered by excessive g-forces, all data was deleted permanently 10 s later. Data were protected from unauthorized access and removal and was only viewable by DriveCam’s staff and site research staff. The site research staff, comprised of a neurologist, neuropsychologist, and occupational therapist specializing in driving evaluation, reviewed the events weekly to ensure no egregious events occurred that would prompt recommendation for a road test or driving cessation. DriveCam staff scored all videos according to a standardized procedure developed and validated by their company for commercial drivers (Myers et al., 2012). DriveCam staff were blind to all clinical information regarding dementia severity.

A commercial GPS datalogger (G2 Tracking Device^TM^, Azuga Inc., San Jose, CA, United States) was plugged into the vehicle’s OBD-II port and data collected every 30 s. This naturalistic driving methodology, termed the Driving Real World In-Vehicle Evaluation System (DRIVES), is a validated method of driving data collection for older adults (Babulal et al., 2016). The device collected data every time the vehicle was driven and recorded adverse driving events (hard braking, sudden acceleration, and speeding) anytime they occurred during a trip, regardless of the 30 s sampling that occurred for other datalogger measures. Speeding was determined based on the datalogger’s GPS, specifically the latitude and longitude and the posted speed limit in the vehicle’s location. The device compared the vehicle’s speed to the posted speed limit and if the driver was going 6 miles per hour or more above the posted speed limit in that area, an occurrence of speeding was recorded.

#### Driving Behavior

A total of 3 months of driving were selected for study for all participants from a larger sample of longitudinal driving. Three months were selected because the cognitively impaired group was enrolled in a driving intervention trial where the first 3 months were monitoring only. Inclusion of only the first 3 months of driving avoids any confounds associated with the intervention. To ensure that we were only analyzing the driving behavior of the study driver, videos captured by other drivers were deleted. Since the driver cannot be identified by the GPS logger, drivers had to be driving their vehicle for at least 75% of the time to be included in the current study. Vehicle use was reported by the study partner (*M* = 94%, range = 75–100). The cognitively normal control group were exclusive drivers of their vehicles (100%). For all groups, any driving events were deleted if they were captured during known times that the study driver was not driving due to illness, travel, etc.

Driving errors captured by video were categorized into the following behaviors and scored according to total demerit points: collisions/near collisions, distractions (food, passengers, cell phone, and other electronic devices), awareness (late response, poor scanning of roadway, and failure to check mirrors), driver conduct (poor judgment, aggressive/reckless), fundamentals (excessive speed for conditions, failure to leave an out, and unsafe lane change), following too close, driver condition (drowsy), traffic violation (rolling stop, failure to stop at stop sign or light, speeding, not on designated roadway, and unsafe/risky behavior). The specific events or problems were graded for safety risk on a 0–10 point demerit scale. A single unsafe driving event could have more than one demerit category, such as judgment error combined with poor awareness of intersection, leading to a combined driving severity rating score for the individual items. Error frequency was used to describe error types. Total demerit points were used to examine correlational relationships between driving events captured with video and GPS logger behavior.

Driving behavior captured by the DRIVES were aggregated from daily trip reports for each vehicle over the course of the participant’s entire participation in the study (up to 5 years). Daily trip data included date, starting and ending latitude and longitude, starting and ending time, distance of trip (miles), trip time (minutes), idling time (minutes), and counts of hard braking, sudden acceleration, and speeding. The first 3 months of a participant’s driving behavior were extracted and were examined per 100 trips, where a trip was defined from “ignition on” to “ignition off.” For example, an excursion from home to the grocery store and back to home without any other stops would be considered two trips. Hard braking, hard acceleration and speeding events per trip were analyzed separately and in combination. As multiple events of a particular type could occur in a single trip, data were analyzed for each event type using a two-step procedure: (a) number of trips per 100 in which such an event occurred and (b) number of events per trip for trips with at least one such event. In addition, other aspects of speeding, such as the duration of speeding episodes, average speed in miles, trip distance and trip time, were analyzed.

### Statistical Analysis

Participants with and without preclinical AD (all CDR = 0) were matched on age and gender to participants with symptomatic AD (CDR > 0). Spearman’s ρ correlational analyses using Bonferroni correction for multiple comparisons were used to examine relationships between driving behavior captured with video and the GPS datalogger. Generalized linear modeling (GLM) techniques were used to determine effects of amyloid and cognitive status on event frequency per 1,000 miles driven. All analyses were carried out using the GLM function in R 3.5.3^1^. Event frequency was modeled via an over-dispersed Poisson distribution with amyloid group (CN/A−, CN/A+, and AD) as the sole model predictor. Exposure differences were accounted for by adjusting for 1,000 miles driven via an offset variable. Point and interval estimates of group effects were estimated in the logarithmic scale as log (Rate Ratios) and then exponentiated. In addition, measures of cognition (MMSE, Trails A and Trails B time) were analyzed as secondary predictors of event frequency.

## Results

**FIGURE 1:**
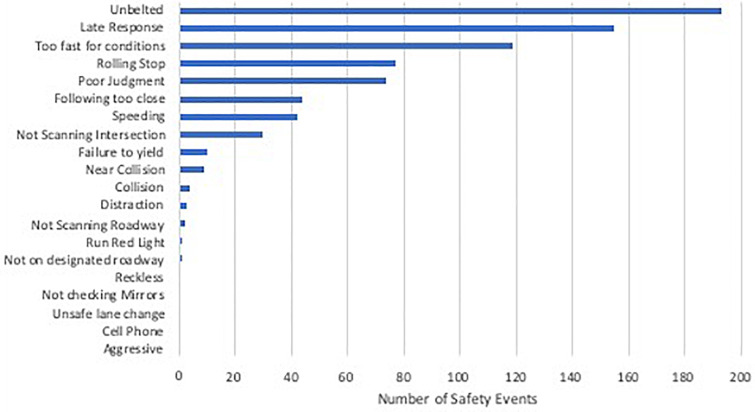
Total number of unsafe driving events video recorded over 3 months of driving in the AD group (*n* = 11).

Demographic characteristics of these older drivers are presented in Table 1. Groups were matched on age (*p* = 0.95), education (*p* = 0.19), and gender. As expected, the early AD group had lower MMSE scores than the cognitively normal groups. When examining the early AD group with the video technology, there were four collisions with objects such as curbs, mail boxes, parked cars, but none with other moving vehicles. The most frequently occurring safety events were driving unbelted, late response, too fast for conditions, rolling stop, poor judgment, following too close, speeding, and failing to scan intersections (see Figure 1).

The next set of analyses included all three groups to examine differences in driving behavior using the DRIVES. Table 2 shows that hard braking events were the most commonly occurring behavior on a per trip basis across all three groups, followed by speeding in the CN/A− group and by hard acceleration in the CN/A+ and early AD groups. Summing across event types, driving events occurred in one in five trips taken by CN/A− or early AD drivers vs. one in eight trips taken by CN/A+. When only considering trips in which adverse driving events occurred (see Table 3), repeat events in the same trip were only common for speeding, with 0.9 more speeding events per trip on average in the CN/A− and early AD groups compared to the amyloid positive group (4.3 vs. 3.4 speeding events).

**TABLE 2:** Adverse driving event rate per 100 trips using DRIVES technology.

**Event Type**	**Group**		
	**CN/A−**	**CN/A+**	**AD**
Hard breaking	11.07	8.13	8.52
Hard acceleration	5.98	4.45	8.52
Speeding	9.81	2.21	5.52
Overall	21.50	12.61	19.42

**TABLE 3:** Adverse driving event count per trip for trips including an event using DRIVES technology.

**Event type**	**Group**		
	**CN/A−**	**CN/A+**	**AD**
Hard breaking	1.27	1.26	1.28
Hard acceleration	1.47	1.26	1.41
Speeding	4.27	3.37	4.30
Overall	3.01	1.85	2.40

To control for driving exposure, driving events were corrected per 1,000 miles driven (see Table 4 for point estimates and 95% confidence intervals for the driving event rate per 1,000 miles driven). After correcting for driving exposure, speeding was the most common event type in both the CN/A− and early AD groups, while hard braking remained the most common event type in the CN/A+. Table 5 shows point estimates and 95% confidence intervals for the ratio of driving events per 1,000 miles driven between (a) the cognitively normal groups (CN/A− vs. CN/A+) and (b) early AD vs. preclinical AD (CN/A+). The preclinical AD group had the lowest rate across all three types of driving events (hard braking, hard acceleration, and speeding), but the differences were especially pronounced for speeding behavior.

**TABLE 4:** Adverse driving rate per 1,000 miles driven (95% Confidence Interval) using DRIVES technology.

**Event type**	**Group**		
	**CN/A−**	**CN/A+**	**AD**
Hard breaking	19.7 (13.9, 27.8)	13.9 (10.1, 19.3)	18.1 (13.2, 25.0)
Hard acceleration	12.3 (3.5, 42.8)	7.6 (3.7, 15.7)	20.0 (8.0, 50.5)
Speeding	58.5 (37.7, 90.6)	10.1 (3.4, 30.4)	39.6 (18.3 86.0)
Overall	90.4 (69.0, 118.4)	31.6 (17.6, 57.0)	77.8 (50.3, 120.3)

**TABLE 5:** Ratios of adverse driving rates per 1,000 miles driven (95% Confidence Interval) using DRIVES technology.

**Event type**	**Group**	
	**CH/Amyloid− vs. CH/Amyloid+**	**AD vs. CH/Amyloid+**
Hard breaking	1.41 (0.88, 2.27)	1.30 (0.83, 2.05)
Hard acceleration	1.62 (0.38, 6.84)	2.64 (0.82, 8.53)
Speeding	5.78 (1.77, 18.90)^++^	3.92 (1.02, 15.10)^+^
Overall	2.86 (1.50, 5.46)^+++^	2.46 (1.18, 5.11)^+^

*^+^Implies *p* < 0.05, ^++^Implies *p* < 0.01, ^+++^Implies *p* < 0.001.*

Spearman correlations were calculated to examine the relationship between error types captured with each technology in the mild AD group. The strongest correlations were between speeding registered by the datalogger and collision/near collisions and fundamentals of driving (i.e., failing to keep an out, too fast for conditions, and failure to yield; rho = 0.81 and 0.87, respectively. Hard breaking and hard acceleration were not consistently related to errors captured by video analysis.

**FIGURE 2:**
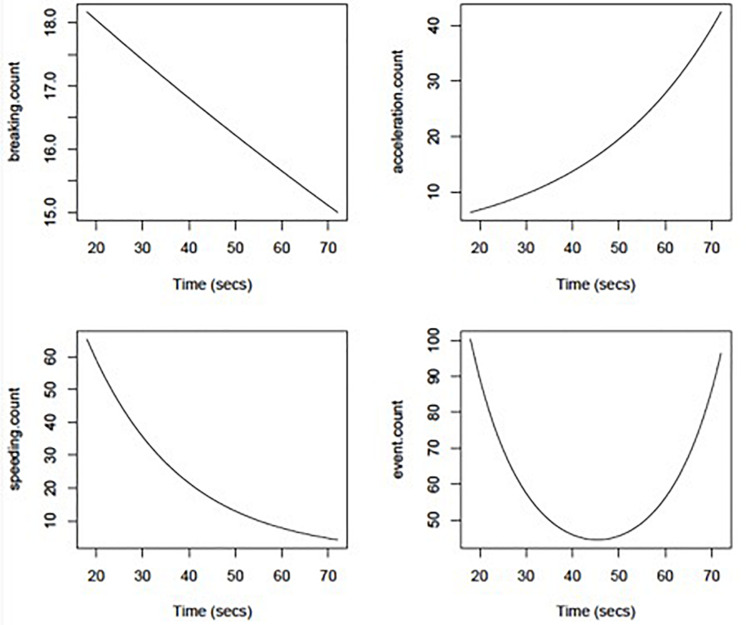
Adverse driving rates per 1,000 miles driven using DRIVES technology as a function of Trail Making Part A duration (in seconds).

Poisson regression models showed no significant effect of MMSE or Trails B time on driving event frequency (count of braking, speeding, and sudden acceleration in a trip). However, Trails A time had significant non-linear effects on overall event frequency (*p* = 0.03) that are depicted graphically in Figure 2. Further analysis of the non-linear effects (e.g., parabolic curve) taken apart showed that the initial drop in driving events was correlated with lower speeding event rates with increasing Trails A time (*p* = 0.03), whereas the later increase in events was related to higher hard acceleration event rates with increasing Trails A time (*p* = 0.09). Hard braking event rates were relatively insensitive to Trails A time (*p* = 0.64).

## Discussion

Two in-vehicle technologies were used in our study to characterize driving errors and behaviors in older adult drivers with preclinical and early symptomatic AD compared to cognitively normal adults without any evidence of AD pathology. In the early AD drivers, g-forced triggered events produced common errors predominantly related to inadequate anticipation of situations, such as late response or driving too fast for the situation. AD drivers also frequently showed errors of judgment and made frequent traffic violations around speeding and responding to road signage (i.e., stop signs and traffic lights). Despite these instances of poor judgment, actual collisions were very rare during the study period. These g-force triggered safety events are consistent with the types of driving events we previously captured with continuous video recording in mild AD drivers compared to cognitively normal older adults (Davis et al., 2012). These findings also confirm prior data that used a semiautomated data reduction method to isolate relevant driving errors from continuous video recorded naturalistic driving to detect cognitive impairment-associated driving behaviors in older adults (Davis et al., 2018; Moharrer et al., 2020). This suggests that an event-based approach, rather than more costly and staff time intensive continuous monitoring of behavior via video, may be a sensitive method to study driving risk in AD.

Global positioning system data logger technology was used to address potential differences in driving behaviors among cognitively normal, preclinical AD, and symptomatic AD. From this data, a distinct pattern of driving behavior emerged among drivers with preclinical AD compared to cognitively normal older adults without evidence of AD and early AD. Specifically, drivers with preclinical AD drove more slowly and had the lowest number of aggressive events over the 3-month period. These data are also consistent with prior work showing that older drivers in the preclinical phase of AD restrict their driving compared to healthy peers (Babulal et al., 2019; Roe et al., 2019).

The current results extend these findings by offering insights into how driving may change across the spectrum of normal aging to symptomatic AD by including a group of mild AD drivers for comparison. Results suggest that in the earliest stage of AD there may be a period of self-regulatory behavior during amyloid accumulation but where cognitive functioning remains unaffected. As the disease progresses and cognition begins to decline with disease progression and neurodegeneration, inhibitory control over more aggressive driving behavior may begin to erode. As such, the AD drivers may revert back to “normal” driving habits including excessive speed. This is consistent with prior work showing that AD drivers whose naturalistic driving was video recorded showed poorer tactical self-regulation behavior and made twice as many critical events as healthy older drivers. They were also three times more likely to be unaware of these events (Paire-Ficout et al., 2018). Unfortunately, the early AD group may need to continue to use compensatory strategies (i.e., cautious driving) to prevent more egregious safety errors and accidents. It is possible that early AD drivers could maintain independence longer by increasing their awareness of driving errors and the provision of compensatory strategies. Prior intervention studies suggest that this may be possible. For example, we showed that a behavioral intervention aimed at correcting the specific driving errors reduced the frequency of driving errors in a group of drivers with early AD (Ott et al., 2017a).

Given that the cognitive processes of attention and executive functioning decline in AD and have been shown to relate to driving errors (Anderson et al., 2012; Papandonatos et al., 2015), we examined the relationship between measures of these constructs and driving events captured by the data logger. In this study, simple psychomotor speed was associated with driving behavior. Specifically, more acceleration events, slower speeds, and more overall events were associated with slower psychomotor speed. This suggests a relationship between cognitive impairment and driving behaviors may be captured with the data logger.

This study utilized two different passive monitoring in-vehicle technologies to understand driving behavior. As such, it was of interest to explore the relationship between behaviors captured with g-force triggered video technology vs. the data logger, as the video provides more context to the behaviors captured with the data logger. Data indicated that some, but not all, event types were highly related. Specifically instances of speeding registered by the datalogger were related to instances of collision/near collisions and errors in driving fundamentals. Hard breaking and hard acceleration were not strongly related to safety errors. These relationships could only be examined in a small sample of early AD participants, and more work will be needed to better understand these findings, but preliminarily, these data support the idea that aggressive events captured with the data logger may indeed reflect risky driving behavior.

There are several limitations to this study. First, this is a small sample of older drivers, and results should be viewed as preliminary and only applicable to the aging population. The CN and early AD participants were recruited from two regionally different locations. It is possible that geographic differences in population density, type of driving, and seasonal weather changes may have impacted the results. The cognitively normal older adult drivers (CDR 0) did not have the video technology installed in their vehicles, so it is unclear how these behaviors may occur in a cognitively normal population or the degree to which amyloidosis might influence this relationship based on these data alone. In addition, it is unclear the degree to which events captured with the GPS data logger correlate with actual unsafe behavior or simply more assertive or effective defensive driving in a healthy population. The strong relationship between video captured safety events and GPS events in the cognitively impaired group would suggest that the GPS captured events reflect actual risky behavior, possibly more erratic driving, in the cognitively impaired group. Lastly, results need be replicated in a larger sample of racially and ethnically diverse older drivers as participants in this study were all non-Hispanic white, which limits generalizability to other diverse populations.

Our results offer preliminary findings that suggest that in-vehicle technology can detect behavioral differences between drivers at different points in the spectrum of normal aging to early AD. With the increase in Advanced Driver Assistance Systems (ADAS) as standard features in vehicles, instrumented vehicle technology may offer a unique opportunity to detect early behavioral change in older adults that could signal increased risk for unsafe driving. These types of technology could be used to identify when an individual may need to start considering driving retirement with instances of unsafe behaviors serving as early markers of cognitive decline. Objective measurement of driving changes, in conjunction with report of driving changes using driving questionnaires could lead to further assessment with an occupational therapist, driving specialist, or monitoring by a healthcare provider. Future research should employ multiple modalities for assessing driving behavior over extended periods of time to obtain a more complete characterization of the aging driver.

## Data Availability Statement

The raw data supporting the conclusions of this article will be made available by the authors, without undue reservation.

## Ethics Statement

The studies involving human participants were reviewed and approved by the Rhode Island Institutional Review Board. The patients/participants provided their written informed consent to participate in this study.

## Author Contributions

JD and GB contributed to data collection, data interpretation, and manuscript preparation. GP conducted statistical analyses. CR provided data management. BO and CR contributed to the interpretation of results and manuscript preparation. EB provided study coordination and data collection. All authors listed have made a substantial, direct and intellectual contribution to the work, and approved it for publication.

## Conflict of Interest

The authors declare that the research was conducted in the absence of any commercial or financial relationships that could be construed as a potential conflict of interest.

## Footnotes

1 https://cran.r-project.org
